# Understanding What Drives Long-term Engagement in Digital Mental Health Interventions: Secondary Causal Analysis of the Relationship Between Social Networking and Therapy Engagement

**DOI:** 10.2196/44812

**Published:** 2023-05-22

**Authors:** Shaunagh O'Sullivan, Niels van Berkel, Vassilis Kostakos, Lianne Schmaal, Simon D'Alfonso, Lee Valentine, Sarah Bendall, Barnaby Nelson, John F Gleeson, Mario Alvarez-Jimenez

**Affiliations:** 1 Centre for Youth Mental Health University of Melbourne Melbourne Australia; 2 Orygen Parkville Australia; 3 Department of Computer Science Aalborg University Aalborg Denmark; 4 School of Computing and Information Systems University of Melbourne Melbourne Australia; 5 Healthy Brain and Mind Research Centre Australian Catholic University Melbourne Australia; 6 School of Behavioural and Health Sciences Australian Catholic University Melbourne Australia

**Keywords:** digital intervention, digital health, youth mental health, psychotic disorders, usage metrics, log data, social networking

## Abstract

**Background:**

Low engagement rates with digital mental health interventions are a major challenge in the field. Multicomponent digital interventions aim to improve engagement by adding components such as social networks. Although social networks may be engaging, they may not be sufficient to improve clinical outcomes or lead users to engage with key therapeutic components. Therefore, we need to understand what components drive engagement with digital mental health interventions overall and what drives engagement with key therapeutic components.

**Objective:**

Horyzons was an 18-month digital mental health intervention for young people recovering from first-episode psychosis, incorporating therapeutic content and a private social network. However, it is unclear whether use of the social network leads to subsequent use of therapeutic content or vice versa. This study aimed to determine the causal relationship between the social networking and therapeutic components of Horyzons.

**Methods:**

Participants comprised 82 young people (16-27 years) recovering from first-episode psychosis. Multiple convergent cross mapping was used to test causality, as a secondary analysis of the Horyzons intervention. Multiple convergent cross mapping tested the direction of the relationship between each pair of social and therapeutic system usage variables on Horyzons, using longitudinal usage data.

**Results:**

Results indicated that the social networking aspects of Horyzons were most engaging. Posting on the social network drove engagement with all therapeutic components (*r*=0.06-0.36). Reacting to social network posts drove engagement with all therapeutic components (*r*=0.39-0.65). Commenting on social network posts drove engagement with most therapeutic components (*r*=0.11-0.18). Liking social network posts drove engagement with most therapeutic components (*r*=0.09-0.17). However, starting a therapy pathway led to commenting on social network posts (*r*=0.05) and liking social network posts (*r*=0.06), and completing a therapy action led to commenting on social network posts (*r*=0.14) and liking social network posts (*r*=0.15).

**Conclusions:**

The online social network was a key driver of long-term engagement with the Horyzons intervention and fostered engagement with key therapeutic components and ingredients of the intervention. Online social networks can be further leveraged to engage young people with therapeutic content to ensure treatment effects are maintained and to create virtuous cycles between all intervention components to maintain engagement.

**Trial Registration:**

Australian New Zealand Clinical Trials Registry: ACTRN12614000009617; https://www.australianclinicaltrials.gov.au/anzctr/trial/ACTRN12614000009617

## Introduction

### Background

Poor engagement with digital mental health interventions is a major challenge in the field [1]. For example, Fleming and colleagues [2] found that there is a 15-day retention rate of only 3.9% and a 30-day retention rate of only 3.3% for the use of mental health apps in the general population and low completion or usage rates beyond 6 weeks (0.5% to 28.6%) in digital interventions targeting depression and anxiety.

A recent development in the field is the personalization of digital mental health interventions to enhance the user experience and engagement [3-6]. These include the use of machine learning algorithms [3,4] and prompts or reminders [5,6] to tailor interventions to individual users needs and patterns of engagement. Another novel example is the use of multicomponent interventions that increase flexibility to personalize and cater for the needs of different individuals, thereby promoting engagement [1,7]. These interventions capitalize on human support to promote engagement and clinical effects and on social media to promote sustained engagement and social support and connectedness [8-10]. Although there are promising indications that additional features in multicomponent digital mental health interventions may increase long-term engagement, little is known about which components are key for efficacy and which components contribute to engagement.

To examine the relationship between engagement and effectiveness, our recent study used a novel method to analyze the relationship between patterns of use of a multicomponent digital platform and treatment effects [11]. Specifically, we characterized the use of social networking and therapeutic aspects of Horyzons, which was an 18-month intervention aiming to improve social functioning, vocational recovery, and relapse prevention in young people recovering from first-episode psychosis (FEP) after receiving 2 years of specialized care [8,12,13]. It included therapy content; a private social network; and peer, clinical, and vocational support. We identified 3 user profiles: (1) low usage, (2) maintained usage of social networking elements, and (3) maintained usage of both social networking and therapeutic elements of the intervention. Results indicated that engagement with the therapy components of the platform was needed for improved outcomes in terms of social functioning, overall psychiatric symptom severity, and negative psychotic symptoms and that engaging with the social networking components of the platform alone was not sufficient to bring about clinical benefits. However, social networking may be key for engagement, as all sustained use of the intervention included use of the social network [11,14]. However, analyses such as those used in this recent study are not designed to measure drivers of engagement and causal relationships between intervention components. Thus, we need novel methods to analyze what drives engagement overall and what drives engagement with the subset of specific components associated with therapeutic mechanisms.

The field of human computer interaction (HCI) utilizes empirical dynamic modeling (EDM) as a nonlinear analysis method to investigate human use of technology from time series data, where interactions with technology change over time and may be bidirectional, and associations can be inferred from the data rather than predetermined hypotheses [15]. Convergent cross mapping (CCM) is a type of analysis based on EDM that tests causal relationships between variables, by establishing if states of the causal variable can be recovered from the time series of the affected variable [16]. Researchers then extended this approach to multiple CCM (MCCM), which accounted for multiple relationships or users of technology in the analysis [17]. As relationships between system use variables can be complex and nonlinear, linear analysis methods and those that make correlation-based inferences are not appropriate to investigate what aspects of usage lead to subsequent usage of intervention components [15].

The field of HCI has started to investigate causal and potentially bidirectional relationships between users and technology using MCCM, to improve and design better interactive technology [17]. It has been suggested that interaction with technology should be considered as a complex dynamic system, whereby our use of a technological system is affected by our mental state and our mental state is affected by our use of a technological system [17]. Usage behavior has previously been analyzed to determine the causal relationship between smartphone application use and emotional states [18]. This study found that application use drives user emotions in most cases but that user emotions also drive some aspects of application use [19]. Determining causality is important so that developers and researchers can take appropriate actions to ensure users access engaging information efficiently and effectively and to maximize the use of all intervention components. Furthermore, if we know what intervention components are linked to better outcomes, we can optimize interventions to enhance engagement with the therapeutic or active ingredients of these interventions.

### Objective

Horyzons is an example of a unique long-term multicomponent digital intervention aiming to maintain long-term treatment effects and engagement. Our previous research found a relationship between improved outcomes and combined use of the therapy and social networking components of Horyzons [11]. However, little is known about whether aspects of the social network aspect drive use with aspects of therapy content or vice versa. This study, therefore, aimed to determine the causal relationship between different aspects of system use and whether use of the social network leads to subsequent use of therapeutic content by applying the MCCM method.

## Methods

### Study Design

Horyzons was a single-blind, 18-month, randomized controlled trial (RCT) conducted with young people recovering from FEP, following 2 years of specialist early intervention treatment. Participants were randomly allocated to either treatment as usual (TAU) or TAU as well as 18 months of access to the Horyzons intervention [12]. Horyzons was underpinned by the moderated online social therapy (MOST) model, which integrates (1) interactive online therapy (“Pathways and Steps”), (2) peer-to-peer online social networking, (3) peer moderation, and (4) expert clinical and vocational support [20].

### Ethics Approval

Ethics approval was obtained from the Melbourne Health Research Ethics Committee (2013.146).

### Participants

Participants included 86 young people allocated to the Horyzons intervention. These participants were recruited after receiving 2 years of specialized care from the Early Psychosis Prevention and Intervention Centre (EPPIC) at Orygen, in Melbourne Australia between October 2013 and January 2017. EPPIC is a specialist FEP service that provides 18 months to 24 months of specialist early intervention for FEP to young people aged 15 years to 24 years [21,22].

Of the participants allocated to the intervention arm of the RCT, 5% (4/86) did not engage with the Horyzons platform. As there was no valid usage data, these participants were excluded from the analysis. The remaining 82 intervention participants were aged between 16 years and 27 years at randomization (mean 21, SD 2.88 years). As participants were recruited following their completion of early intervention treatment, this accounts for the age range extending to 27 years old. According to the Horyzons RCT eligibility criteria [13], participants were required to meet the criteria for an FEP disorder or mood disorder with psychotic features according to the Diagnostic and Statistical Manual of Mental Disorders 4th Edition [23], to have not been treated with antipsychotic medication for more than 6 months before attending EPPIC, and to have demonstrated remission of positive symptoms of psychosis for 4 weeks or more at the time of enrolment in the Horyzons study, as measured by the Positive and Negative Syndrome Scale [24].

### System Usage Metrics

System usage metrics were extracted from the Horyzons online platform for each user for each day of their trial involvement (range: 282-528 days per user), resulting in a total of 47,060 cases of user interaction. See Table 1 for an overview of the metrics representing aspects of daily usage of the intervention’s therapeutic and social components.

**Table 1:** System usage variables extracted from the Horyzons platform.

Variable type	Variables
Therapy-related variables (number of)	Steps^a^ started Pathways^b^ started Actions^c^ done Visits to suggested content^d^ Visits to messages^e^ Visits to therapy^f^
Social networking–related variables (number of)	News feed^g^ posts News feed comments Likes made Reactions^h^ made

^a^Steps refer to the intervention modules.

^b^Pathways refer to a collection of intervention modules related to a topic (eg, anxiety).

^c^Actions refer to behavioral activities aiming to translate learning into behavior.

^d^Suggested content refers to therapeutic content recommended by clinical moderators.

^e^Messages refer to a private message section, where moderators could contact participants directly.

^f^Visiting therapy refers to visiting the home page of the therapy component of the intervention.

^g^The news feed refers to the social network.

^h^Reactions refer to short support messages in response to a post (eg, thinking of you).

We categorized *pathways* based on their therapeutic targets, which included understanding psychosis; identifying early warning signs to prevent relapse; identifying and exercising personal strengths; promoting social connections and positive emotions; and managing stress, anxiety, and depression. As a means to increase engagement, *Pathways* were distilled into shorter, interactive *Steps* (eg, illustrating how to respond empathically to others [to foster positive connections]). See Multimedia Appendix 1 for an example of a *Step* on Horyzons. Each *Step* was partnered with an *Action* or “*Do It*,” which was designed to support the translation from learning into behavioral change (eg, suggestions on how to exercise empathy in specific contexts). Expert clinical moderators could also recommend *Pathways,* *Steps, Actions*, and *Talk it Outs* (described in the following paragraphs) that were personally appropriate to the young person via a private message, which would appear as a notification in the user’s dashboard. Furthermore, users could visit the therapeutic components of Horyzons without completing any therapeutic content (eg, viewing what *Pathway* and *Step* were currently allocated to them).

The social network was moderated and led by peer workers. MOST peer workers were young people who identified as having a lived experience of mental ill-health and who had been employed and trained to offer support and guidance to others on the MOST platform. The social network was designed for participants to communicate and foster a sense of social support. Participants were able to post comments*,* or “like,” “respond,” or “react,” to comments posted by other young people. A set of limited *reactions* (or *emojis*) was available to communicate social support in response to posts (eg, “I get you,” “thinking of you”). Finally, the *Talk It Out* function allowed young people to suggest relevant discussion topics to take place in a separate forum moderated by the peer workers. The function was informed by an evidence-based problem-solving framework [25]. Participants received notifications when other users communicated on the social network. Participants also received private messages when a moderator contacted them directly via the platform. See Multimedia Appendix 2 for an example of a newsfeed post with *likes* and *reactions* on the Horyzons social network.

### Statistical Analyses

#### MCCM

MCCM was used to determine the causal and potentially bidirectional relationship between the aforementioned system usage metrics. For example, we tested whether usage of certain aspects of the social network leads to usage of certain therapy components or vice versa. This is a new methodology that extends beyond existing CCM methods and was adapted from that of Van Berkel and colleagues [17].

CCM is a core component of the EDM approach, which is a set of methods designed to characterize and test causality in complex dynamic systems, such as users interacting with technology over time [17]. CCM was developed for use with time series data to distinguish causality from correlation [26]. CCM considers time series data from a complex and dynamic systems perspective and investigates the relationships between variables in a system that is not completely random [17]. CCM is considered to be a novel and suitable method for studying human behavior when it is modeled on a complex system [19,27,28]. Analyses were conducted using the “rEDM” R package [29,30]. The 4 steps of the method are described in the following paragraphs.

#### Identify the Optimum Value for E (Embedding Dimension)

The optimum embedding dimension (E) was identified between each pair of system usage variables tested between the social and therapy components of the intervention. This was done using simplex projection, which is the most direct projection technique and recommended for evaluating embedding dimensions for EDM [29]. The method uses time delay embedding on a single variable (*y*) to generate a complex system reconstruction, by using history information of another variable (*x*), and determines how much information about *x* has been encoded into *y* [31]. It is justified by the embedding theorem by Takens [31-33], where, if *x* influences *y*, the historical values of *x* can be recovered from *y*. It then applies the simplex projection algorithm to make forecasts, and the highest E value is selected [19].

#### Test for Nonlinearity

As CCM is a nonlinear approach, it was also important to test whether system usage evolves in a nonlinear way. The rEMD package uses S-maps to characterize the degree of nonlinearity in the time series, by using the E chosen from the previous step of simplex projection and then estimating a linear map that uses the E-dimensional points on a manifold’s surface (ie, dimension of Euclidean space) to predict the future [17,26]. The data are nonlinear if the maximum forecasts skill is greater than 0.

#### Convergent Cross Mapping

CCM was then used to identify potential causal links between pairs of system usage variables for each user. Using this approach, pairs of variables are mapped to each other using the nearest neighbors of each point on the E-dimensional manifolds (ie, the causal effects of *x* on *y* are determined by how well *y* cross maps *x*). When the number of points on the manifold increases, the nearest neighbors tend to become nearer, which improves predictions if the variables are causally linked (ie, convergence) [17].

#### Multiple Convergent Cross Mapping

The previous step analyzed the causal relationship between variables for each individual user, which works well when trying to understand the dynamics of an individual ecosystem (ie, 1 user). This step extends upon that approach using a geometric approach and summarizes the results from multiple CCM analyses. This is called MCCM and enables us to obtain insights into the behavior of the entire study population [17]. Effect sizes were calculated using Pearson *r* correlation (along with standard deviations), with scores ranging from 0 to 1.

## Results

### Overview

The causal and potentially bidirectional relationship between usage of social networking and therapeutic aspects of Horyzons were investigated, to determine whether social networking usage leads to engagement with therapy content or vice versa.

**Figure 1:**
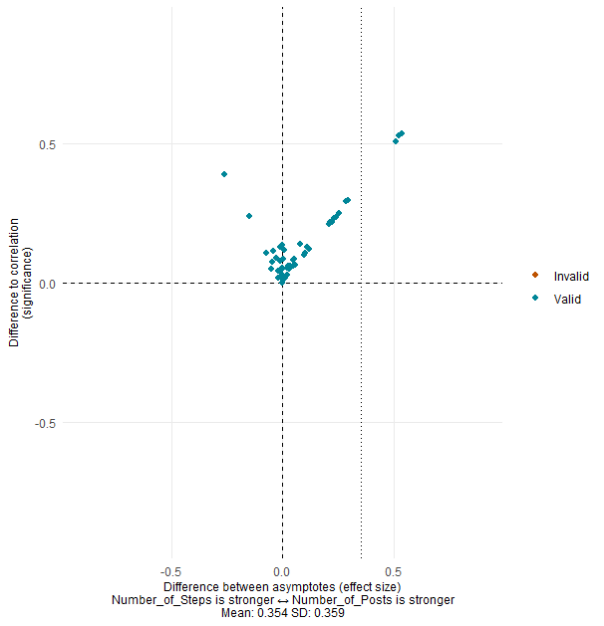
Visual representation of the direction of the causal relationship between completing therapeutic steps and posting on the social network.

A visual representation of results for each pair of variables for all combinations of variables described in Table 1 (eg, steps started [therapy] versus posts made [social networking]) is available in Multimedia Appendices 3-6, and an example of a graph for 1 pair of variables can be seen in Figure 1. Each participant in the graph is represented by a dot. For each participant, the CCM algorithm determines whether variable 1 (completing therapy steps) is driven by variable 2 (posting on the social network) or vice versa.

The direction and magnitude of effects are represented by the position of the dots on the x axis. If the dot is to the left of the y axis, it indicates that the effect is in a certain direction (ie, completing therapy steps drives posting on the social network). If the dot is to the right, it indicates that the effect is in the other direction (ie, posting on the social network drives completing therapy steps; see Figure 1).

The distance from the x axis indicates how strong the forecasting ability is compared with a plain correlation, and the dashed vertical line indicates the mean effect size of causality across all participants. Any red dots that are present on graphs should be ignored as they represent invalid participant data, due to CCM not being able to provide power beyond that of a simple correlation [19].

### Directional Relationship Between Posting on the Social Network and Therapy Engagement

MCCM was used to determine the causal relationship between social networking and therapy engagement. Table 2 shows the mean effect size of causality (Pearson *r*) among the analyzed variable pairs. These results indicate that posting on the social network drives all aspects of therapy engagement, including completing steps (*r*=0.354), completing pathways (*r*=0.154), completing actions (*r*=0.055), visiting suggested content (*r*=0.295), visiting messages (*r*=0.284), and visiting therapy (*r*=0.360). A visual inspection of these results can be found in Multimedia Appendix 3, where the prevalence of points to the right of most of the graphs indicates that posts made on the social network lead to usage of therapeutic content for most participants. However, there are also some participants with dots to the left of the graphs, indicating that their usage of therapeutic content leads to posting on the social network. Overall, however, we conclude that posts drive therapy engagement as indicated by the vertical dotted line to the right of all graphs in Multimedia Appendix 3.

**Table 2:** Effect sizes (and standard deviations) for causality between therapy usage and social network usage.

Therapy usage	Social network usage			
	Posts made, *r* (SD)	Comments made, *r* (SD)	Likes made, *r* (SD)	Reactions made, *r* (SD)
Steps started	0.354 (0.359)^a^	0.161 (0.326)^a^	0.166 (0.315)^a^	0.653 (0.230)^a^
Pathways started	0.154 (0.467)^a^	–0.047 (0.418)^b^	–0.058 (0.397)^b^	0.484 (0.446)^a^
Actions completed	0.055 (0.437)^a^	–0.142 (0.393)^b^	–0.148 (0.397)^b^	0.390 (0.422)^a^
Suggested content visited	0.295 (0.373)^a^	0.120 (0.315)^a^	0.110 (0.327)^a^	0.582 (0.316)^a^
Messages visited	0.284 (0.359)^a^	0.112 (0.306)^a^	0.087 (0.338)^a^	0.589 (0.288)^a^
Therapy visited	0.360 (0.350)^a^	0.177 (0.307)^a^	0.167 (0.296)^a^	0.649 (0.230)^a^

^a^Social network usage drives therapy usage.

^b^Therapy usage drives social network usage.

### Directional Relationship Between Commenting on the Social Network and Therapy Engagement

On the other hand, comments made on the social network and therapy engagement demonstrated a bidirectional relationship, whereby some aspects of therapy engagement such as completing pathways (*r*=–0.047) and completing actions (*r*=–0.142) drove the commenting on the social network, while commenting on the social network drove other aspects of therapy engagement such as completing steps (*r*=0.161), visiting suggested content (*r*=0.120), visiting messages (*r*=0.112), and visiting therapy (*r*=0.177; see Table 2). A visual inspection of these results can be found in Multimedia Appendix 4.

### Directional Relationship Between Liking Posts on the Social Network and Therapy Engagement

Similarly, likes made on the social network and therapy engagement have a bidirectional relationship, whereby some aspects of therapy engagement such as completing pathways (*r*=–0.058) and completing actions (*r*=–0.148) drove liking a post on the social network, while liking a post on the social network drove other aspects of therapy engagement such as completing steps (*r*=0.166), visiting suggested content (*r*=0.110), visiting messages (*r*=0.087), and visiting therapy (*r*=0.167; see Table 2). A visual inspection of these results can be found in Multimedia Appendix 5.

### Directional Relationship Between Reacting to Posts on the Social Network and Therapy Engagement

Finally, our MCCM analysis showed that reacting to posts made on the social network drove all aspects of therapy engagement, including completing steps (*r*=0.653), completing pathways (*r*=0.484), completing actions (*r*=0.390), visiting suggested content (*r*=0.582), visiting messages (*r*=0.589), and visiting therapy (*r*=0.649; see Table 2). A visual inspection of these results can be found in Multimedia Appendix 6.

## Discussion

### Principal Findings

This was the first study to use a novel modeling technique like MCCM to determine the causal relationship between different aspects of use of a long-term multicomponent digital intervention (Horyzons) to improve social functioning, improve vocational recovery, and prevent relapse in FEP. We found that posting on the social network and reacting to posts on the social network led to engaging with all aspects of therapy (including completing steps, pathways, and actions and visiting suggested content, messages, and therapy). We also found that commenting on the social network and liking posts made on the social network led to most aspects of therapy engagement (including completing steps and visiting suggested content, messages, and therapy). In other words, young people’s use of the social network was found to increase therapy use on Horyzons. Conversely, we found a bidirectional relationship between therapeutic and social components, whereby completing actions and pathways led to commenting on posts and liking posts on the social network, even though these aspects of social network usage led to engagement with all other measured aspects of therapy.

Overall, we found that usage of social networking aspects of Horyzons drove engagement with the therapeutic aspects in most cases. Therefore, we need to further understand what drives engagement with the social network [34], but it is also important to understand how the social network drives engagement with digital interventions in general (eg, self-determination theory [SDT] can inform engagement) and what aspects of the social network can drive engagement with therapy specifically (eg, social validation and social comparison). Previous research has also indicated that the use of prompts, texts, and emails may enhance engagement with digital interventions in general [6,35]. Horyzons was underpinned by SDT to inform engagement, which predicts that support for the following 3 basic psychological needs promotes motivation for behavior change: (1) autonomy (having a choice about how to behave), (2) competence (being able to make changes to achieve desired outcomes), and (3) relatedness (feeling accepted in one’s social environment) [36]. According to SDT, participants’ relatedness needs have to be supported by the system for engagement to occur, which was incorporated into supportive accountability. However, future qualitative research could further explore why the use of social networking aspects of interventions lead people to then engage with therapy components and explore whether positive feedback or experiences from the social network encouraged people to further engage with therapy content.

It is then essential to engineer social networks in digital mental health interventions in ways that promote engagement with key therapeutic ingredients. This is important, as recent research has found that those who maintain use of both social networking and therapy components of an intervention display improved outcomes, but use of social networks alone may not lead to improvements [11]. Interestingly, this study found that the social network was useful to promote overall engagement as well as engagement with key therapeutic ingredients of the platform. This is needed, as sustained engagement with both social and therapy content has been associated with improved social functioning, negative symptoms, and overall symptoms [11]. Therefore, although the social network may not be therapeutic in isolation, it works as part of a multicomponent digital mental health intervention and has significant value in promoting engagement with key therapeutic ingredients of the platform, especially with the use of reactions leading to use of all therapy components (*r*=0.39-0.65). Sustained engagement is needed in digital mental health interventions, especially in long-term relapse prevention interventions like Horyzons targeting long-term outcomes. Horyzons showcased elements of therapy on the social network, which possibly contributed to the findings in this study. However, this could be improved by adding additional engaging social networking components, and social networks more generally could aim to improve outcomes in this way in the future, by showcasing therapy on their social networks and providing more personalized suggestions for therapy via the social network.

In particular, the largest effect sizes were observed for reacting to posts on the social network, which led to usage of all assessed aspects of therapy (*r*=0.39-0.65). This is an interesting finding, as reacting to posts on the social network was deemed passive use of the social network [11]. However, passive use of social media has been associated with increased anxiety in adolescents, but this was not linked to therapeutic sites specially [37]. Therefore, although passive elements of the social network may be the most engaging, it is important that they lead young people to therapeutic content to bring about clinical benefits. Furthermore, the reactions used in Horyzons were more active than reactions on traditional social networks, as they were based on social support and validation (eg, thinking of you, I get you). It may be the case that these types of reactions, which were engineered to promote safe and meaningful connections, may be a better driver of engagement with therapy than more passive reactions, providing insight into types of novel design solutions that can enhance engagement with key therapeutic ingredients.

However, it should be noted that this study also indicated that some aspects of therapy led to social network use, potentially generating synergistic effects between social networking and therapy usage once the transition to therapy is made. This, in turn, could promote engagement with both aspects of the intervention where usage of the social network and key therapeutic ingredients reinforce each other, leading to sustained engagement. For example, once the transition is made to engage with therapy, there may be a synergistic effect whereby social networking drives therapy engagement but also some aspects of therapy usage that drive social networking (eg, in this study, we found that the number of actions completed drives commenting on the social network). Learning from others and sharing experiences may lead to increased motivation to engage with the platform and with therapy more specifically and, in turn, lead to people sharing their therapy journeys with each other [38]. In fact, the MOST platform (which Horyzons was based on) was created to do this by including *talking points* in the interactive therapy content, where a user could comment on the therapy content that was then shared to the social network, thus promoting back and forth cycles between both elements. The findings from this study partly support this hypothesis but indicate that more research is needed to optimize the synergistic use of both elements, which are likely needed for long-term engagement with digital interventions and is a challenge in the field [38].

This study also suggests we need to establish how different components of a platform interact to promote engagement and drive positive outcomes, and the MCCM method has enormous potential to optimize platforms to be more engaging and more therapeutic. Future research should focus on how to optimize the platform to make the social network more therapeutic, promote more engagement with therapy, and generate back and forth virtuous cycles between both intervention components. Predictors of engagement could also be explored (eg, using prompts) and applied to aspects of the intervention that drive engagement with therapeutic content or key active ingredients (eg, a notification asking a participant if they would like to react to a new post on the social network) [39]. Establishing engagement with therapeutic social networks can promote engagement with other therapeutic aspects of digital mental health interventions, which have displayed high rates of attrition to date. This, in turn, could lead to optimization of the synergistic effects of intervention components in multicomponent digital interventions to improve outcomes.

### Limitations

However, a number of limitations must also be noted. The sample size for this study was small, comprising 82 young people, so findings should be interpreted with caution. Although this was the first causal analysis exploring how the use of certain intervention components leads to subsequent use of other components, this study only tested the direction of the relationship between therapy and social networking components of a digital mental health intervention. It is possible that other multicomponent digital mental health interventions may include additional components, and some may be more engaging than a social network. It is also possible that multicomponent interventions not focused on mental health may have different engaging components, and future research should consider this.

### Conclusions

To date, digital interventions have shown significant limitations with long-term participant engagement, a necessary ingredient toward bringing about long-term improvements in mental health. Multicomponent digital interventions that incorporate social networks have been proposed as a means to promote long-term engagement and tackle elusive long-term targets such as relapse prevention, which are rarely targeted by digital interventions. In a previous study, we identified that sustained use of both therapy and social networking components of a digital mental health intervention led to improved outcomes for young people recovering from FEP. This study adds to this knowledge by showing that the social network is possibly a necessary ingredient for long-term engagement and positive outcomes but not sufficient when used alone. Our study highlights the possible value of the social network in promoting engagement with key therapeutic ingredients of the intervention. Future research should aim to confirm these findings with a larger sample size and, if confirmed, could focus on how to optimize the social network to be more therapeutic, to more widely promote engagement with key therapeutic ingredients of the intervention, and to create virtuous cycles between use of the social network and therapeutic content.

## Appendix

Multimedia Appendix 1 Horyzons step: how to flourish.

Multimedia Appendix 2 Horyzons social network.

Multimedia Appendix 3 Social network posts drive therapy engagement.

Multimedia Appendix 4 Comments made and therapy engagement have a bidirectional relationship.

Multimedia Appendix 5 Likes made and therapy engagement have a bidirectional relationship.

Multimedia Appendix 6 Reactions drive therapy engagement.

## Abbreviations

CCM: convergent cross mapping
EDM: empirical dynamic modeling
EPPIC: Early Psychosis Prevention and Intervention Centre
FEP: first-episode psychosis
HCI: human computer interaction
MCCM: multiple convergent cross mapping
MOST: moderated online social therapy
RCT: randomized controlled trial
SDT: self-determination theory
TAU: treatment as usual
