# Supplementary figures and images for: Understanding What Drives Long-term Engagement in Digital Mental Health Interventions: Secondary Causal Analysis of the Relationship Between Social Networking and Therapy Engagement
Source: JMIR Ment Health. 2023 May 22;10:e44812. doi: 10.2196/44812 (PMC10242471; doi:10.2196/44812)

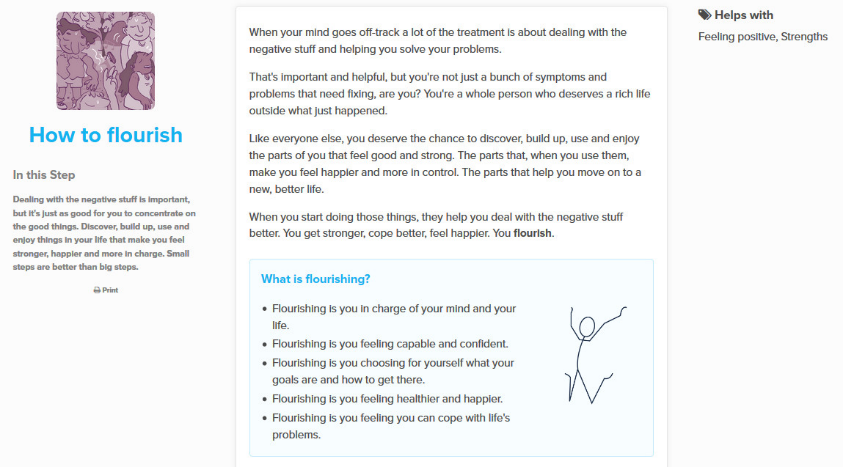

Supplement: Multimedia Appendix 1 [file mental_v10i1e44812_app1.png]

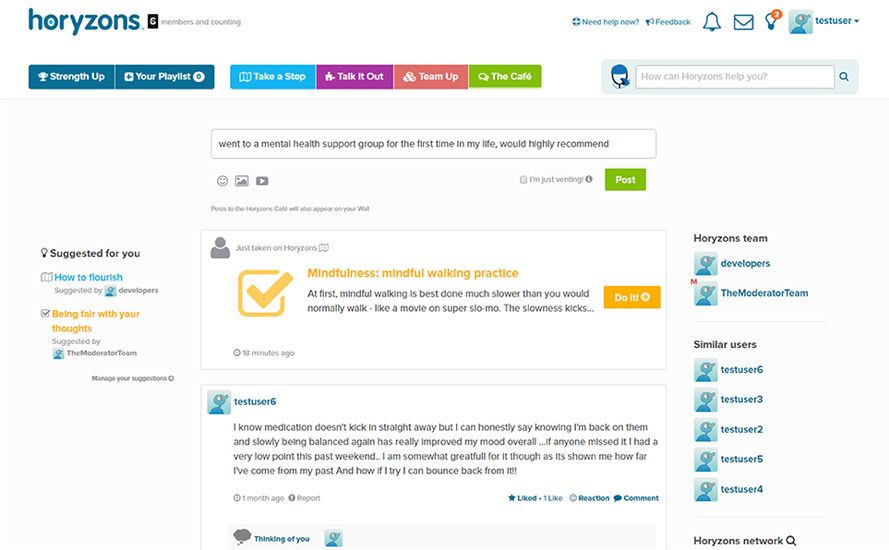

Supplement: Multimedia Appendix 2 [file mental_v10i1e44812_app2.png]

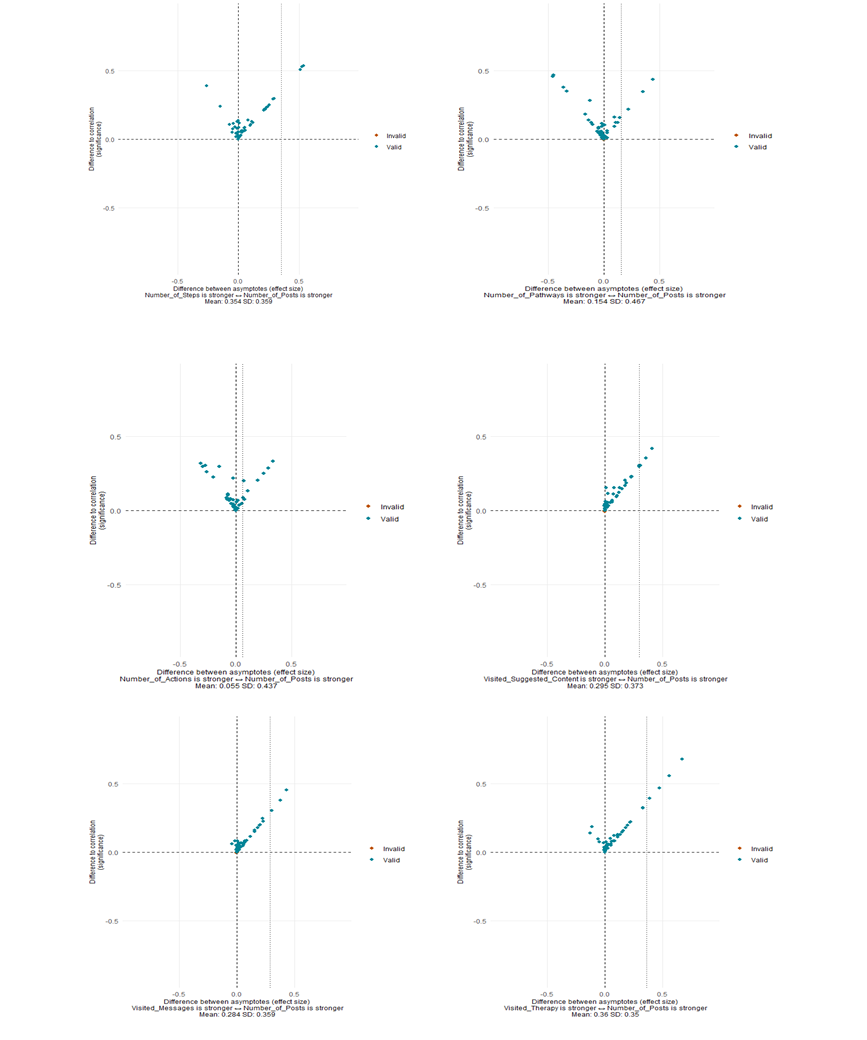

Supplement: Multimedia Appendix 3 [file mental_v10i1e44812_app3.png]

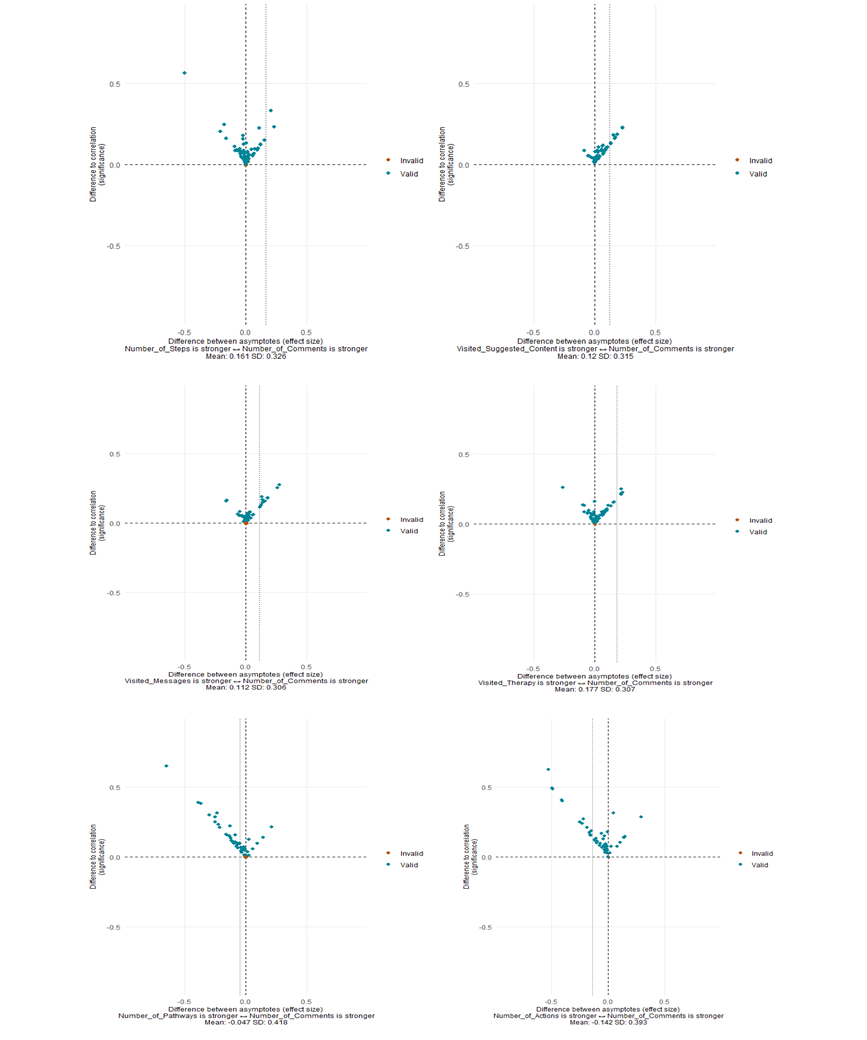

Supplement: Multimedia Appendix 4 [file mental_v10i1e44812_app4.png]

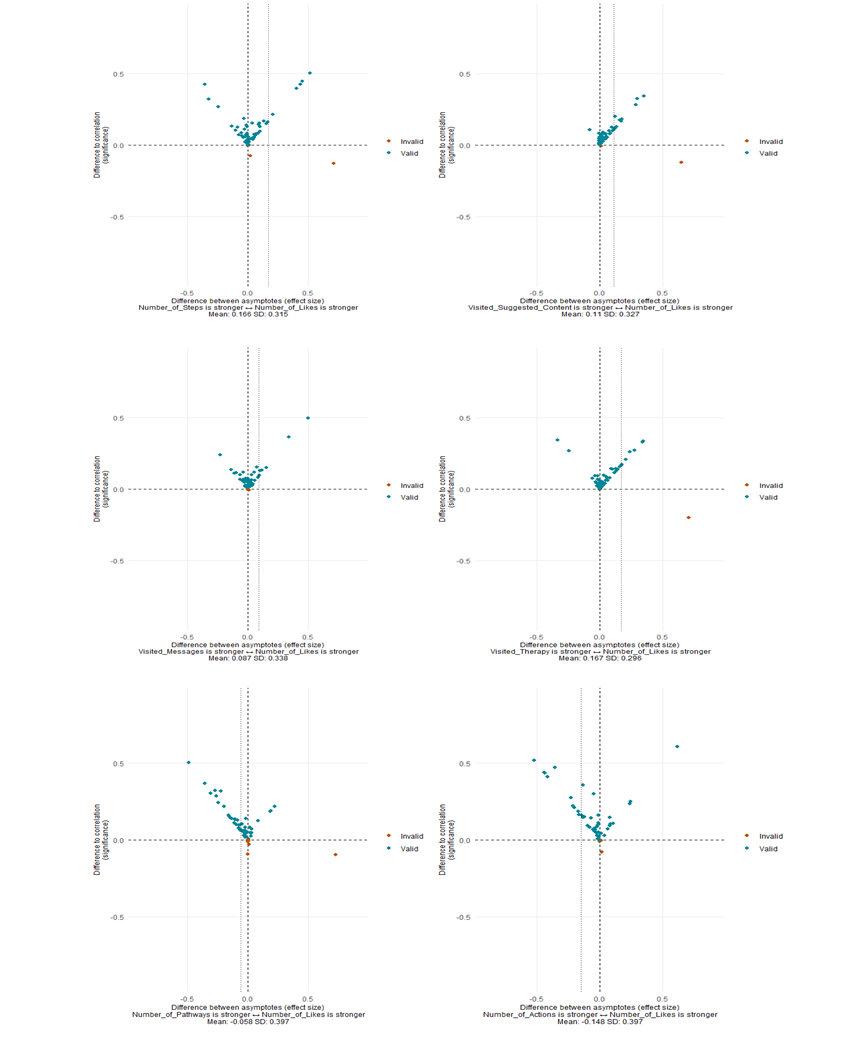

Supplement: Multimedia Appendix 5 [file mental_v10i1e44812_app5.png]

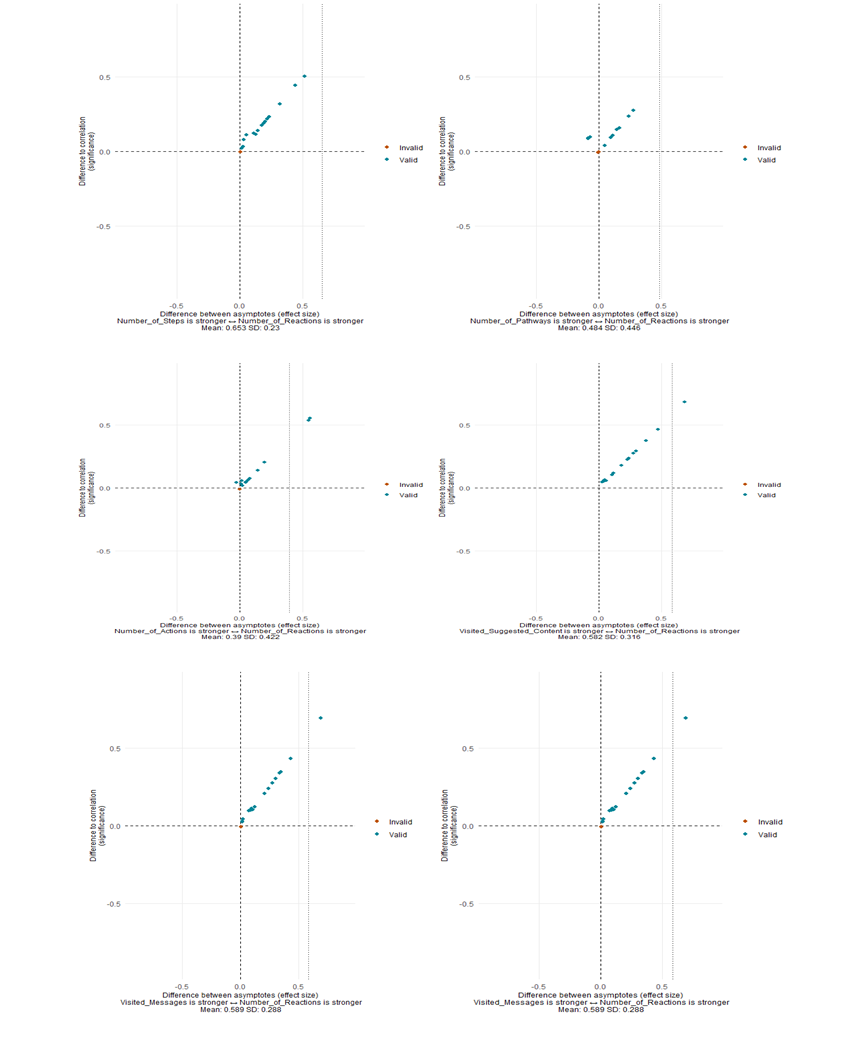

Supplement: Multimedia Appendix 6 [file mental_v10i1e44812_app6.png]
